# Functional Polymorphisms of Matrix Metalloproteinases 1 and 9 Genes in Women with Spontaneous Preterm Birth

**DOI:** 10.1155/2014/171036

**Published:** 2014-10-28

**Authors:** Nina Pereza, Ivana Pleša, Ana Peterlin, Žiga Jan, Nataša Tul, Miljenko Kapović, Saša Ostojić, Borut Peterlin

**Affiliations:** ^1^Department of Biology and Medical Genetics, Faculty of Medicine, University of Rijeka, B. Branchetta 20, 51000 Rijeka, Croatia; ^2^Clinical Institute of Medical Genetics, Department of Gynaecology and Obstetrics, University Medical Center Ljubljana, Šlajmerjeva 3, 1000 Ljubljana, Slovenia; ^3^Division of Obstetrics and Gynecology, Department of Perinatology, University Medical Center Ljubljana, Šlajmerjeva 3, 1000 Ljubljana, Slovenia

## Abstract

*Objective*. The aim of this study was to investigate the association of functional *MMP-1*-1607 1G/2G and *MMP-9*-1562 C/T gene polymorphisms with spontaneous preterm birth (SPTB; preterm birth with intact membranes) in European Caucasian women, as well as the contribution of these polymorphisms to different clinical features of women with SPTB. *Methods and Patients*. A case-control study was conducted in 113 women with SPTB and 119 women with term delivery (control group). Genotyping of *MMP-1*-1607 1G/2G and *MMP-9*-1562 C/T gene polymorphisms was performed using the combination of polymerase chain reaction and restriction fragment length polymorphism methods. *Results*. There were no statistically significant differences in the distribution of neither individual nor combinations of genotype and allele frequencies of *MMP-1*-1607 1G/2G and *MMP-9*-1562 C/T polymorphisms between women with SPTB and control women. Additionally, these polymorphisms do not contribute to any of the clinical characteristics of women with SPTB, including positive and negative family history of SPTB, gestational age at delivery, and maternal age at delivery, nor fetal birth weight. *Conclusion*. We did not find the evidence to support the association of *MMP-1*-1607 1G/2G and *MMP-9*-1562 C/T gene polymorphisms with SPTB in European Caucasian women.

## 1. Introduction

Preterm birth (PTB) is a common complication of pregnancy and an important perinatal health problem, occurring in 9.6% of all births worldwide [1]. It is defined as childbirth before 37 completed weeks or 259 days of gestation and accounts for almost 70% of neonatal mortality and morbidity [1–3]. Additionally, children born prematurely are at an increased risk of long-term health complications [1, 3].

Preterm birth can be the consequence of three conditions, including preterm premature rupture of membranes (PPROM), medical indications, or preterm labor with intact membranes (spontaneous PTB; SPTB) [3, 4]. The latter accounts for almost 50% of all cases of PTB [1, 3, 4]. Although the causes of SPTB are unknown, epidemiologic data point to a potential contribution of genetic factors [2, 3, 5, 6]. Firstly, women with a personal or family history have an increased risk of PTB compared with women in the general population [3, 7]. Additionally, twin studies suggest heritability for PTB ranges from 20% to 40% [8]. Finally, substantial differences have been determined in the rate of PTB across different racial and ethnic groups [9]. The contribution of genetic variability to PTB was evaluated for several candidate genes, divided into two groups. The first group comprises genes encoding products involved in host response to infection and inflammation, whereas the products of genes in the second group participate in extracellular matrix (ECM) remodeling.

Although it is unknown whether the initial signal for parturition derives from the fetus or the mother, extensive ECM degradation occurs in fetal membranes, cervix, and decidua during the final weeks of pregnancy, allowing the rupture of fetal membranes, cervix dilatation, and placental detachment from uterus [10, 11]. Degradation of the ECM is controlled by matrix metalloproteinases (MMP), a family of 23 zinc-dependent endopeptidases [12]. The levels of MMPs in the cervix, lower uterine segment, amniotic fluid, fetal membranes, and maternal plasma increase at labor, indicating their precise spatial and timely regulation is needed to prevent PTB [11, 13–16].

Among the MMPs, MMP-1, and MMP-9 have been extensively examined in women with PTB. Most studies reported alterations of* MMP-1* and* MMP-9* gene expression in terms of increased levels in serum, amniotic fluid, fetal membranes, cervical fibroblasts, and cervical mucus plug in women with PTB compared to women with term delivery [11, 15–20]. Although the causes of this altered gene expression are unknown, functional polymorphisms located in* MMP-1* and* MMP*-*9* promoter regions might be a contributing factor. An insertion-deletion polymorphism of a single guanine (1G or 2G) is located at nucleotide-1607 in the* MMP-1* gene promoter, and the presence of the additional guanine leads to up to a fourfold increased promoter activity [21, 22]. Additionally, a single nucleotide polymorphism at nucleotide-1562 in the* MMP-9* gene promoter results from the substitution of cytosine (C) with thymine (T), which increases promoter activity due to the loss of the binding site for an unknown transcription repressor [23].

Two previous studies evaluated the potential role of the* MMP-9*-1562 C/T gene polymorphism as a factor of predisposition to PPROM in African American and Chinese women, and one study included non-Hispanic white women with PTB, which was not classified into PPROM and SPTB [24–26]. However, the association of* MMP-1*-1607 1G/2G and* MMP-9*-1562 C/T with SPTB in European Caucasian women has never been tested. Therefore, in view of the important roles of MMP-1 and MMP-9 in the pathogenesis of PTB, the aim of this study was to investigate the association of functional* MMP-1*-1607 1G/2G and* MMP-9*-1562 C/T gene polymorphisms with SPTB in European Caucasian women. Furthermore, we analyzed the contribution of these polymorphisms to different clinical features of women with SPTB.

## 2. Materials and Methods

### 2.1. Subjects

We conducted a case-control study in order to evaluate the potential association of* MMP-1*-1607 1G/2G and* MMP-9*-1562 C/T gene polymorphisms with SPTB. A total of 113 women with SPTB and 119 control women were included in the study. Demographic and clinical data of women with SPTB and their newborn children were collected in accordance with the data set for genetic epidemiology studies into PTB [3]. Data were collected by means of a self-developed questionnaire which was completed by investigators.

All women with PTB had singleton pregnancies following natural conception and spontaneous initiation of PTB before 37 weeks of gestation. Gestational age was determined by last menstrual period and confirmed by ultrasound in the first trimester. In cases where estimated gestational age from the last menstrual period and ultrasound differed for more than 7 days, gestational age was changed according to ultrasound measurement in the first trimester. The initial study group consisted of 118 women with preterm birth; however five women with PPROM were excluded from genetic analysis. None of the women had known risk factors for PTB, including diabetes, hypertension, kidney disease, autoimmune conditions, allergic diseases, birth canal infections, in vitro fertilization, and complications of pregnancy. Furthermore, none of the live born had congenital anomalies or evidence of infection. Additional maternal and newborn characteristics are shown in Table 1.

For each woman with SPTB one woman of the same age and parity with term delivery of singleton baby after uncomplicated pregnancy was included in the study.

All women from the study and control groups were Caucasians and delivered at Division of Perinatology at Department of Obstetrics and Gynaecology, University Medical Center in Ljubljana, Slovenia. Written informed consent was obtained from all participants. The study was approved by the Slovenian National Medical Ethics Committee.

### 2.2. Methods

#### 2.2.1. DNA Extraction

Genomic DNA of all women was extracted from peripheral blood leukocytes by standard procedure using commercially available kit (Qiagen FlexiGene DNA kit, Qiagen GmbH, Hilden, Germany). Extracted DNA was stored at −20°C.

#### 2.2.2. Genotype Analysis

Genotyping of* MMP-1*-1607 1G/2G and* MMP-9*-1562 C/T gene polymorphisms was performed using the combination of polymerase chain reaction (PCR) and restriction fragment length polymorphism (RFLP) methods. Primers, PCR-RFLP reaction conditions, as well as the expected sizes of PCR products, and restriction fragments were described in detail in our previous study [27]. PCR amplification was carried out in a thermal cycler (Mastercycle personal, Eppendorf, Hamburg, Germany). The restriction digestion of PCR products was carried out following the manufacturer's recommended conditions. PCR products and restriction fragments were separated using electrophoresis on 3% agarose gels stained with GelRed (Olerup SSP, Saltsjöbaden, Sweden) and the product bands were visualized under ultraviolet light.

#### 2.2.3. Statistical Analysis

Statistical analyses were performed using Statistica for Windows, version 10 (StatSoft, Inc., Tulsa, OK, USA). In addition, statistical power and sample size were calculated using DSS Researcher's Toolkit (https://www.dssresearch.com/toolkit/spcalc/power_p2.asp), whereas odds ratios (OR) and 95% confidence intervals (95% CI) were calculated using MedCalc for Windows, version 12.7.7. (MedCalc Software, Mariakerke, Belgium). Hardy-Weinberg equilibrium was calculated using Simple Hardy-Weinberg Calculator-Court Lab (Washington State University College of Veterinary Medicine, Pullman, WA, USA).

Differences in genotype and allele frequencies between women with SPTB and control women were determined using Pearson's chi square (*X* ^2^) test. The association of* MMP-1*-1607 1G/2G and* MMP-9*-1562 C/T gene polymorphisms with SPTB was determined by calculating ORs and their 95% CIs according to different genetic models (dominant, recessive, codominant). Distribution of numerical variables was tested using Kolmogorov-Smirnov test. One-way analysis of variance (ANOVA) was used for comparison of age and fetal birth weight means between* MMP-1*-1607 1G/2G genotypes, whereas Student's *t*-test was used for comparison of age and fetal birth means between* MMP-9*-1562 C/T genotypes. Statistical significance was set at *P* values <0.05.

## 3. Results

Genotype distributions in women with SPTB and control women were in Hardy-Weinberg equilibrium for both* MMP-1*-1607 1G/2G and* MMP-9*-1562 C/T gene polymorphisms (data not shown). Our study had 100% and 90% power to detect a twofold increase in* MMP-1*-1607 1G allele and* MMP-9*-1562 T allele, respectively.

The distribution of* MMP-1*-1607 1G/2G and* MMP-9*-1562 C/T genotypes and alleles frequencies in women with SPTB and control women are shown in Table 2. The association between the two polymorphisms and the risk of SPTB according to dominant, recessive, and codominant genetic models is shown in Table 3. There were no statistically significant differences in the distribution of genotype and allele frequencies of either polymorphism between women with SPTB and control women. Additionally, there was no association between* MMP-1*-1607 1G/2G and* MMP-9*-1562 C/T genotypes and alleles and the risk of SPTB under any genetic model. Finally, no significant differences were observed in the distribution of any combination of* MMP-1*-1607 1G/2G and* MMP-9*-1562 C/T genotypes between women with SPTB and control women (data not shown).

We further evaluated the association between the* MMP-1*-1607 1G/2G and* MMP-9*-1562 C/T genotypes and alleles and various clinical features of women with SPTB. However, there were no statistically significant differences in the distribution genotype and allele frequencies of either polymorphism between women with positive and negative family history of SPTB (Tables 4 and 5), as well as between women according to gestational age at delivery (Tables 6 and 7). Furthermore, there were no statistically significant differences between* MMP-1*-1607 1G/2G and* MMP-9*-1562 C/T genotypes and maternal age at delivery (*P* = 0.856 and *P* = 0.807, respectively; full data not shown) nor fetal birth weight (*P* = 0.850 and *P* = 0.612, respectively; full data not shown).

## 4. Discussion

In the present study, we investigated for the first time whether there was an association of the functional* MMP-1*-1607 1G/2G and* MMP-9*-1562 C/T gene polymorphisms with SPTB in European Caucasian women. Differences in the distribution of individual and combined genotype and allele frequencies of these polymorphisms between women with SPTB and control women did not reach statistical significance. Moreover, there was no association between* MMP-1*-1607 1G/2G and* MMP-9*-1562 C/T genotypes and alleles and the risk of SPTB according to dominant, recessive, and codominant genetic models.

To the best of our knowledge, the association between SPTB in European Caucasian women and* MMP-1*-1607 1G/2G and* MMP-9*-1562 C/T gene polymorphisms was not previously investigated. However, two studies analyzed the association between the* MMP-9*-1562 C/T gene polymorphism in African American and Chinese women with PPROM [24, 25]. Another study was performed in non-Hispanic white women with PTB, which was not classified into PPROM and SPTB, therefore not allowing an adequate comparison with our results [26]. Chinese women carrying the CT and TT genotype had 5.31 times increased risk of PTB than those carrying the CC genotype (95% CI = 1.07–26.44) [25]. Similarly, the* MMP-9*-1562 C/T gene polymorphism was associated with PTB in non-Hispanic white women; however, the authors did not specify which genotype is the potential risk genotype [26]. In contrast, this polymorphism was not associated with PPROM in African American women, which is comparable to our results in European Caucasian women with SPTB [24]. Although no previous studies investigated the potential role of* MMP-1*-1607 1G/2G gene polymorphism in SPTB in women, significant association was found between the 2G allele and PPROM in the offspring of African American women with PTB [21]. Furthermore, the* MMP-1* gene promoter containing the 2G allele had a twofold increased activity compared with the 1G allele in amnion cells, indicating that it could be a risk factor for PTB.

Another aim of this study was to determine the contribution of* MMP-1*-1607 1G/2G and* MMP-9*-1562 C/T gene polymorphisms to different clinical features of women with SPTB, which could alter the risk of SPTB. First, we analyzed the distribution of genotype and allele frequencies in women whose mother and/or sibling(s) had SPTB and in those with a negative family history. It is well established that women with a positive family history have an increased risk of PTB compared with women in the general population [3, 7, 9]. Also, women who were themselves born prematurely have an increased risk of PTB [7]. In the present study, we did not determine any statistically significant differences in* MMP-1*-1607 1G/2G and* MMP-9*-1562 C/T genotype and allele frequencies between women with a positive and negative family history of SPTB. Second, we investigated whether the genotype and allele frequencies of* MMP-1*-1607 1G/2G and* MMP-9*-1562 C/T gene polymorphisms differ between women according to different gestational age at delivery. PTB is usually classified into extreme (<28 weeks), severe (28–31 weeks), moderate (32-33 weeks), and near term (34–37) [28]. Due to our sample size, we classified women into two categories, moderate preterm (34–37 weeks) and severe preterm (<33 weeks), but there were no statistically significant differences in the distribution of* MMP-1*-1607 1G/2G and* MMP-9*-1562 C/T genotype and allele frequencies between these two SPTB subgroups. Third, older maternal age is associated with PTB, although it is unknown whether maternal age is an independent risk factor for PTB or a risk marker that influences PTB in association with other risk factors [29]. However, in this study, the mean age at delivery did not differ between individual* MMP-1*-1607 1G/2G and* MMP-9*-1562 C/T genotypes in women with SPTB. Finally, we compared the mean birth weight between individual maternal* MMP-1*-1607 1G/2G and* MMP-9*-1562 C/T genotypes but found no differences between maternal genotypes and offspring size at birth.

Although the results of our study indicate the lack of association between* MMP-1*-1607 1G/2G and* MMP-9*-1562 C/T gene polymorphisms and SPTB, further studies are needed to evaluate the role of these, as well as other* MMP* gene polymorphisms in different populations. The onset of labor involves a sequence of events that are the consequence of ECM degradation, including softening and ripening of the cervix, weakening of the fetal membranes, and uterine contractions [10, 13]. The MMPs have a crucial role in all of these processes, MMP-1 enabling the first step of fibrillar collagen cleavage, after which other MMPs, including MMP-2 and MMP-9, further degrade collagen fragments [30]. During normal gestation, MMP-1 and MMP-9 are found in the amniotic fluid and fetal membranes and their levels of expression increase during normal and preterm birth, favoring ECM degradation [13–15, 31, 32]. This increase in* MMP-9* gene expression possibly contributes to ECM degradation in the fetal membranes and placenta, facilitating fetal membrane rupture and placental detachment at labor [15]. The highest enzymatic activity of MMP-9 occurs at the contact region of fetal and maternal parts, indicating the importance of MMP-9 in separation of the placenta from the uterus during delivery [33]. Additionally, placental chorionic villus genes affect the initiation of parturition through altered processing of cell surface molecules by MMP-1 [34].

There are several limitations to this case-control study. For example, we analyzed only maternal genotypes, which do not allow us to draw conclusions on fetal contribution to SPTB nor the potential interaction between maternal and fetal factors to pregnancy outcome. Another limitation is the relatively small sample size. Nevertheless, this study has substantial strengths, such as the inclusion of women with spontaneous PTB only, which according to guidelines for genetic research of PTB increases the homogeneity in the study group and offers increased sensitivity to detect differences in genetic epidemiology studies of PTB [3]. Moreover, we included only women with a standard clinically defined SPTB, had sufficient power analysis, and used peripheral blood samples for DNA analysis.

## 5. Conclusions

In conclusion, we did not find the evidence to support the association of* MMP-1*-1607 1G/2G and* MMP-9-*1562 C/T gene polymorphisms with SPTB in European Caucasian women.

## Figures and Tables

**Table 1:** Characteristics of women with SPTB and their newborn children.

Maternal characteristics^a^	
Maternal age at delivery (years)^b^	30 [18–41]
Gestational age at delivery, *n* (%)	
Severe preterm <33 weeks	29 (26.9)
Moderate preterm 34–37 weeks	79 (73.1)
Smoking during pregnancy, *n* (%)	
Yes	11 (10.2)
No	97 (89.8)
PTB in mother and/or sibling(s), *n* (%)	
Yes	33 (30.6)
No	75 (69.4)
Parity, *n* (%)	
1	65 (60.2)
≥2	43 (39.8)

Newborn characteristics^c^	

Mean birth weight (grams)^b^	2325 [260–3915]
Congenital anomalies, *n*	0
Evidence of infection, *n*	0

^a^Available for 108 women with PTB.

^b^Data is presented as mean and range in parenthesis.

^c^Available for 108 newborn children of women with PTB.

**Table 2:** Genotype and allele frequencies of *MMP-1*-1607 1G/2G and *MMP-9*-1562 C/T gene polymorphisms in women with SPTB and control women.

Gene polymorphism	SPTB (*N* = 113) *n* (%)	Controls (*N* = 119) *n* (%)	Chi square	*P* value
*MMP-1*-1607				
2G2G	24 (21.2)	30 (25.2)		
1G2G	66 (58.4)	64 (53.8)	0.63	0.731
1G1G	23 (20.4)	25 (21.0)		
2G	114 (50.4)	124 (52.1)	0.13	0.721
1G	112 (49.6)	114 (47.9)		
*MMP-9*-1562				
CC	86 (76.1)	83 (69.7)		
CT	27 (23.9)	36 (30.3)	1.18	0.276
TT	0 (0)	0 (0)		
C	199 (88.0)	202 (84.9)	1.00	0.318
T	27 (12.0)	36 (15.1)		

**Table 3:** The association of genotypes and alleles of *MMP-1*-1607 1G/2G and *MMP-9*-1562 C/T gene polymorphisms with SPTB according to dominant, recessive, and codominant genetic models.

Gene	Genetic model	SPTB women versus control women	
		OR (95% CI)	*P*
*MMP-1*-1607	Dominant		
	1G1G + 1G2G versus 2G2G	1.25 (0.68–2.30)	0.475
	Recessive		
	1G1G versus 1G2G + 2G2G	0.96 (0.51–1.81)	0.902
	Codominant		
	1G1G versus 2G2G	1.15 (0.53–2.51)	0.725
	1G1G versus 1G2G	0.89 (0.46–1.73)	0.736
	2G2G versus 1G2G	0.78 (0.41–1.47)	0.435
	Alleles		
	1G versus 2G	1.07 (0.74–1.54)	0.721

*MMP-9*-1562	Dominant		
	CC + CT versus TT	0.95 (0.02–48.27)	0.979
	Recessive		
	CC versus CT + TT	1.38 (0.77–2.47)	0.277
	Codominant		
	CC versus TT	1.03 (0.02–52.82)	0.986
	CC versus CT	1.38 (0.77–2.47)	0.277
	TT versus CT	1.33 (0.02–69.00)	0.986
	Alleles		
	C versus T	1.31 (0.77–2.24)	0.319

**Table 4:** Genotype and allele frequencies of *MMP-1*-1607 1G/2G and *MMP-9*-1562 C/T gene polymorphisms in women with SPTB with positive and negative family history of SPTB.

Gene polymorphism	SPTB in family^a^ (*N* = 33) *n* (%)	Isolated SPTB (*N* = 75) *n* (%)	Chi square	*P* value
*MMP-1*-1607				
2G2G	8 (24.2)	15 (20.0)	0.33	0.849
1G2G	18 (54.6)	45 (60.0)		
1G1G	7 (21.2)	15 (20.0)		
2G	34 (51.5)	75 (50.0)	0.04	0.837
1G	32 (48.5)	75 (50.0)		
*MMP-9*-1562				
CC	23 (69.7)	60 (80.0)	1.37	0.242
CT	10 (30.3)	15 (20.0)		
TT	0 (0)	0 (0)		
C	56 (84.8)	135 (90.0)	1.19	0.276
T	10 (15.2)	15 (10.0)		

^a^In mother and/or sibling(s).

**Table 5:** The association of genotypes and alleles of *MMP-1*-1607 1G/2G and *MMP-9*-1562 C/T gene polymorphisms with familial and isolated SPTB according to dominant, recessive, and codominant genetic models.

Gene	Genetic model	SPTB in family versus isolated SPTB	
		OR (95% CI)	*P*
*MMP-1*-1607	Dominant		
	1G1G + 1G2G versus 2G2G	0.78 (0.29–0.07)	0.620
	Recessive		
	1G1G versus 1G2G + 2G2G	1.08 (0.39–2.92)	0.885
	Codominant		
	1G1G versus 2G2G	0.87 (0.25–3.03)	0.833
	1G1G versus 1G2G	1.17 (0.41–3.33)	0.774
	2G2G versus 1G2G	1.33 (0.48–3.69)	0.579
	Alleles		
	1G versus 2G	0.94 (0.53–1.68)	0.837

*MMP-9*-1562	Dominant		
	CC + CT versus TT	0.44 (0.01–22.84)	0.686
	Recessive		
	CC versus CT + TT	0.57 (0.23–1.46)	0.245
	Codominant		
	CC versus TT	0.39 (0.01–20.15)	0.639
	CC versus CT	0.57 (0.23–1.46)	0.245
	TT versus CT	1.48 (0.03–80.40)	0.849
	Alleles		
	C versus T	0.62 (0.26–1.47)	0.279

**Table 6:** Genotype and allele frequencies of *MMP-1*-1607 1G/2G and *MMP-9*-1562 C/T gene polymorphisms in women with SPTB according to gestational age at delivery.

Gene polymorphism	<33 weeks (*N* = 29) *n* (%)	34–37 weeks (*N* = 79) *n* (%)	Chi square	*P* value
*MMP-1*-1607				
2G2G	6 (20.7)	17 (21.5)	0.29	0.864
1G2G	18 (62.1)	45 (57.0)		
1G1G	5 (17.2)	17 (21.5)		
2G	30 (51.7)	79 (50.0)	0.05	0.822
1G	28 (48.3)	79 (50.0)		
*MMP-9*-1562				
CC	23 (79.3)	60 (75.9)	0.13	0.714
CT	6 (20.7)	19 (24.1)		
TT	0 (0)	0 (0)		
C	52 (89.7)	139 (88.0)	0.12	0.732
T	6 (10.3)	19 (12.0)		

**Table 7:** The association of genotypes and alleles of *MMP-1*-1607 1G/2G and *MMP-9*-1562 C/T gene polymorphisms with SPTB depending on gestational age at delivery according to dominant, recessive, and codominant genetic models.

Gene	Genetic model	<33-week SPTB versus 34–37-week SPTB	
		OR (95% CI)	*P*
*MMP-1*-1607	Dominant		
	1G1G + 1G2G versus 2G2G	0.59 (0.18–1.89)	0.377
	Recessive		
	1G1G versus 1G2G + 2G2G	0.81 (0.21–3.12)	0.762
	Codominant		
	1G1G versus 2G2G	0.57 (0.12–2.73)	0.481
	1G1G versus 1G2G	0.95 (0.23–3.87)	0.940
	2G2G versus 1G2G	1.67 (0.49–5.62)	0.410
	Alleles		
	1G versus 2G	0.77 (0.37–1.61)	0.492

*MMP-9*-1562	Dominant		
	CC + CT versus TT	0.19 (0.00–9.96)	0.412
	Recessive		
	CC versus CT + TT	5.73 (0.72–45.57)	0.099
	Codominant		
	CC versus TT	0.24 (0.00–12.78)	0.485
	CC versus CT	5.73 (0.72–45.57)	0.099
	TT versus CT	16.33 (0.23–1148.0)	0.198
	Alleles		
	C versus T	5.01 (0.65–38.37)	0.121
